# Galectins and Gliomas

**DOI:** 10.1111/j.1750-3639.2009.00270.x

**Published:** 2010-01

**Authors:** Marie Le Mercier, Shannon Fortin, Véronique Mathieu, Robert Kiss, Florence Lefranc

**Affiliations:** 1 Laboratory of Toxicology; Institute of Pharmacy; Université Libre de Bruxelles (ULB)Brussels, Belgium; 2 Department of Neurosurgery; Erasme University Hospital; Université Libre de Bruxelles (ULB)Brussels, Belgium

**Keywords:** Galectin, Glioma, Migration, Angiogenesis, Resistance

## Abstract

Malignant gliomas, especially glioblastomas, are associated with a dismal prognosis. Despite advances in diagnosis and treatment, glioblastoma patients still have a median survival expectancy of only 14 months. This poor prognosis can be at least partly explained by the fact that glioma cells diffusely infiltrate the brain parenchyma and exhibit decreased levels of apoptosis, and thus resistance to cytotoxic drugs. Galectins are a family of mammalian beta-galactoside-binding proteins characterized by a shared characteristic amino acid sequence. They are expressed differentially in normal vs. neoplastic tissues and are known to play important roles in several biological processes such as cell proliferation, death and migration. This review focuses on the role played by galectins, especially galectin-1 and galectin-3, in glioma biology. The involvement of these galectins in different steps of glioma malignant progression such as migration, angiogenesis or chemoresistance makes them potentially good targets for the development of new drugs to combat these malignant tumors.

## GLIOMAS: AN OVERVIEW

Gliomas account for more than 50% of all primary brain tumors and are by far the most common primary brain tumor in adults (71, 73, 77). Gliomas include tumors that are composed predominantly of astrocytes (astrocytomas), oligodendrocytes (oligodendrogliomas), ependymal cells (ependymomas) or a mixture of various glial cells (eg, oligoastrocytomas) (77). The World Health Organization grading system classifies gliomas into grade I to IV based on the degree of malignancy, as determined by histopathological criteria. Grade I gliomas are generally well circumscribed and behave in a benign fashion, whereas grade II through IV gliomas are malignant and diffusely infiltrate the brain (77). Among gliomas, astrocytomas are the most common and are comprised of pilocytic astrocytomas (grade I), diffuse astrocytomas (grade II), anaplastic astrocytomas (grade III) and glioblastoma (grade IV) (77). Glioblastomas are characterized by a very dismal prognosis (74, 113). Glioblastoma patients have a median survival expectancy of only 14 months when on the current standard treatment of surgical resection to the extent feasible, followed by adjuvant radiotherapy plus temozolomide chemotherapy, given concomitantly with and after radiotherapy (73, 74, 87).

Malignant gliomas are associated with such dismal prognoses because glioma cells can actively migrate through the narrow extracellular spaces in the brain, often traveling relatively long distances, making them elusive targets for effective surgical management (16, 33, 47, 71). Additionally, after surgical resection and adjuvant treatment of malignant gliomas, the residual cancer cells peripheral to the excised lesion give rise to a recurrent tumor that in more than 90% of cases develops immediately adjacent to the resection margin (33, 46, 71).

Clinical and experimental data have also demonstrated that invasive malignant glioma cells show a decrease in proliferation rate and a relative resistance to apoptosis as compared with the highly dense cellular center of the tumor, and this may contribute to their resistance to conventional pro-apoptotic chemotherapy and radiotherapy (33, 46, 71, 72). One way to potentially overcome resistance to apoptosis is to decrease the migration of malignant glioma cells in the brain, which should then theoretically restore a level of sensitivity to cytotoxic drugs (54, 64, 72, 82).

## GALECTINS: AN OVERVIEW

Galectins are a structurally related family of animal lectins defined by two properties: (i) an affinity for β-galactoside sugars; and (ii) a sequence homology (2, 3, 19, 75). This consensus sequence corresponds to the carbohydrate-recognition domain (CRD), which is a beta sandwich of about 135 amino acids long and is responsible for β-galactoside binding (2, 3, 19, 75). To date 15 galectins have been characterized; they are numbered according to the chronology of their discovery (galectin-1 to galectin-15) (2, 3, 19, 75). The galectins known so far have either one or two CRDs within a single polypeptide chain, and each CRD is not associated with other types of well-defined protein domains. The mono-CRD galectins can be biologically active as monomers (galectin-5, -7, -10) or as homodimers (galectin-1, -2, -11, -13, -14, -15); the bi-CRD galectins (galectin-4, -6, -8, -9, 12) are active as monomers and might also associate into oligomers (69, 70). Galectin-3, a mono-CRD galectin, is unique in that it contains a short proline, glycine and tyrosine rich N-terminal domain fused onto the CRD that therefore allows the formation of oligomers (69, 70). Galectins show a high level of evolutionary conservation, whereby members of this family are present in organisms from nematodes to mammals (49).

Galectins can segregate into multiple cell compartments. Although these proteins lack the signal sequence that would be required for secretion through the classical secretory pathway, some galectins show extracellular localization, suggesting that they are secreted through a non-classical pathway (51, 92, 116). Galectins are present both inside and outside cells. They function extracellularly by interacting with cell surface and extracellular matrix (ECM) glycoproteins and intracellularly by interacting, in a carbohydrate-independent manner, with cytoplasmic and nuclear proteins (24, 42, 75, 101). They play a role in a wide range of processes, including cell adhesion, regulation of cell growth, apoptosis, embryonic development and immune processes-like inflammation (13, 24, 43, 44, 75, 88, 97, 101, 114).

A large amount of experimental evidence has been reported to support the important roles of galectins in cancer biology (19, 23, 24, 75, 115), including tumor angiogenesis (56, 65, 123–125), tumor immune escape (96, 106, 112) and cancer cell migration (35, 45, 53, 86, 134). In the current review, we focus our attention on the biological roles exerted by galectins in gliomas.

## GALECTINS THAT COULD BE IMPLICATED IN GLIOMA BIOLOGY

The group of A. Raz was among the first to demonstrate a relationship between galectin expression and the malignant potential of tumors in the central nervous system (9). Indeed, they have shown that the expression level of galectin-3 was significantly associated with astrocytic tumor grade (9). In contrast, Gordower *et al* found that the level of galectin-3 expression significantly decreases in the majority of astrocytic tumors from low to high grade. However, they also observed that some highly malignant tumor cell clones expressed high amounts of galectin-3 (36). Numerous other studies have also confirmed that astrocytic tumors express high levels of galectin-3 (61, 91, 119, 121, 122). With regard to the other types of glioma, Bressalier *et al* have reported that galectin-3 was not expressed in oligodendrogliomas or ependymomas (9). In another study, galectin-3 expression was shown to be significantly higher in glioblastomas and pilocytic astrocytomas than in oligodendrogliomas, anaplastic oligodendrogliomas and diffuse astrocytomas (91). Finally, it was reported that galectin-3 was expressed in oligodendrocytes, endothelial cells and macrophages/microglial cells in areas of solid tumor growth (22). In this study, Deininger *et al* showed that significantly fewer galectin-3 positive oligodendroglioma cells and macrophages/microglial cells were detected in grade II oligodendrogliomas than in grade III anaplastic oligodendrogliomas. On the contrary, significantly more galectin-3 positive endothelial cells were detected in grade II than in grade III oligdendrogliomas (22). Moreover, in an attempt to reconcile the conflicting results published on galectin-3 expression in human gliomas, Strik *et al* have used immunohistochemistry to identify the cellular origin and extent of galectin-3 positivity in glioma samples (119). They have shown in this study that galectin-3 was expressed in neoplastic astrocytes, macrophages/microglial cells, endothelial cells and some B- and T-lymphocytes. They also pointed out that galectin-3 positivity was considerably influenced by tumor-infiltrating macrophages (119). The expression level of galectin-3 seems then to be highly dependent on non-tumor cells such as endothelial cells or macrophages/microglial cells. This feature can thus partly explain the conflicting results that have been published on galectin-3 expression in human gliomas (22, 119).

The regulation of galectin-3 expression by Runx-2 has been recently suggested to contribute to the malignant progression of glial tumor (128). Runx2 is a member of the Runx family of transcription factors expressed in a variety of human glioma cells, whose expression pattern in these cells strongly correlates with that of galectin-3, but not with that of other galectins (128). Knockdown of Runx2 was shown to be accompanied by a reduction in both galectin-3 mRNA and protein levels by at least 50%, dependent on the glial tumor cell line tested (128).

The role of galectin-1 in glioma biology was first suggested by Yamaoka *et al*(131) and Gunnersen *et al*(39). They have analyzed the mRNA expression of galectin-1 by northern blot in glioma specimens and glioma cell lines. Increased expression of galectin-1 mRNA was shown to correlate with increased malignancy in human astrocytic tumors ranging from low-grade astrocytomas to malignant gliomas (131). However, no statistical analysis was made (131). Two studies from our own group using clinical samples have shown that galectin-1 is expressed in all glioma types and that the level of galectin-1 expression correlates directly with the grade of the astrocytic tumor (10, 105). Specifically, we quantitatively determined (by computer-assisted microscopy) the immunohistochemical expression of galectin-1 in 220 gliomas, including 151 astrocytic, 38 oligodendroglial and 31 ependymal tumors (105). Our data revealed the expression of galectin-1 in all human glioma types with no striking variation in levels among astrocytic, oligodendroglial and ependymal tumors; the level of galectin-1 expression within astrocytic tumors, however, significantly correlated with tumor grade (105). Furthermore, expression levels of galectin-1 in high-grade astrocytic tumors from patients with short-term survival periods were significantly higher than those in tumors from patients with long-term survivals (105).

Very little is known about the expression of other galectins in brain tumors. A reverse transcription polymerase chain reaction analysis has shown that galectin-1, -2, -4, -7, -8 and -9 are expressed in normal human brain (111). In another study, Lahm *et al* have examined the expression of a panel of galectins, including galectin-1, -2, -3, -4, -7, -8 and -9, in eight glioma cell lines (62). Galectin-1, -3 and -8 were the most abundantly expressed in all the cell lines. Galectin-2 was expressed in only one cell line, galectin-4 and -9 were expressed weakly in three cell lines, and no evidence for the presence of galectin-7 mRNA was found among any of the cell lines (62).

## THE INTERACTIONS BETWEEN GALECTINS AND INTEGRINS

Galectins are components of the ECM. The ECM comprises all secreted soluble and insoluble molecules found within the extracellular fluid of the extracellular space. The ECM is not only a static scaffolding for tissue organization but it is involved as well in many regulatory functions like modulation of migration, guidance of axonal growth, synapse formation and cell proliferation. Several reviews have already addressed an in-depth analysis of glioma ECM. We thus cite these reviews without commenting on them, keeping in mind that the current review aims to analyze the roles of galectins in glioma biology. These reviews include the pioneering work of J.T. Rutka (107–109) among others (5, 15, 34, 47, 63, 71, 87, 103, 129).

As emphasized by Uhm *et al*(127), integrins are cell-surface receptors that mediate the physical and functional interactions between a cell and its surrounding ECM. Integrins consist of two non-covalently associated transmembrane glycoprotein subunits alpha and beta, both of which contribute to the binding of ECM components. To date 18 different α-subunits and 8 different β-subunits have been identified, which associate to form 25 recognized αβ heterodimers (48). The specific alpha or beta chains that constitute the integrin receptor determine the repertoire of ECM proteins to which a specific integrin may bind. Moreover, many integrin ligands exhibit a specific three-amino acid sequence labeled arginine–glycine–aspartate (RGD), a sequence that is present in most ECM components (48). Although classically the role ascribed to integrins has been that of anchoring cells to the ECM, the functions of integrins greatly exceed that of mere cell adhesion (127). Within this multifaceted role, integrins have been shown to be molecular determinants of glioma invasion (5, 6, 18, 90, 110).

**Figure 1:**
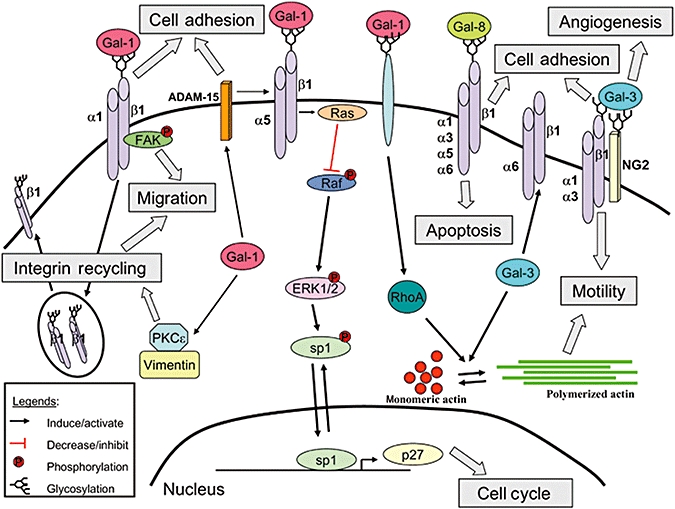
*Galectins, integrins and cell migration*. The interaction of galectins with integrins modulates cell migration as well as other processes. Galectin-1 (Gal-1) interacts with the β1 integrin subunit inducing the phosphorylation of FAK, which modulates cell migration (85). Binding of Gal-1 to integrin is involved in cell adhesion (83). Moreover, Gal-1 was shown to regulate the expression of the protein ADAM-15 that is involved in integrin-mediated adhesion (12). Gal-1 also induces growth inhibition via its interaction with α5β1 (26). This interaction results in the inhibition of the Ras–MEK–ERK pathway and the consecutive transactivation of Sp1, which induces p27 transcription (26). In addition, Gal-1 is involved in the PKCε/vimentin controlled trafficking of integrin β1, a process that is important for cell migration (28). However, it is not known with which molecule(s) Gal-1 is interacting, or in which intra- or extracellular location this interaction is taking place in order to initiate this signaling. Finally, Gal-1 is also involved in cell motility via the Gal-1-induced expression of RhoA and the alteration of the polimerization of the actin cytoskeleton (11) Once again, the receptor to which Gal-1 bind to initiate this signaling is not known. Galectin-3 (Ga-3) regulates cell adhesion via binding to α1β1 (94). Gal-3 also forms a complex with α3β1 and the proteoglycan NG2 (31). This interaction regulates endothelial cell motility and angiogenesis. Finally, Gal-3 has been shown to regulate the expression of integrin α6β1 and actin cytoskeleton organization (20). However, it is not known with which molecule(s) Gal-3 is interacting to initiate this signaling. Galectin-8 (Gal-8) interacts with several integrins including α1β1, α3β1, α5β1 and α6β1. These interactions are involved in cell adhesion and apoptosis (40). Abbreviations: ERK = extracellular signal-regulated kinase; FAK = focal adhesion kinase; MEK = MAP kinase/extracellular signal-regulated kinase kinase (MAPK/ERK Kinase); PKCε = protein kinase C epsilon.

Galectins and integrins closely interact when modulating cell adhesion and/or cell migration. For example, Moiseeva *et al* have shown that galectin-1 interacts with the integrin β1 subunit in vascular smooth muscle cells (85) (Figure 1). Via its direct binding to β1 integrins (without cross-linking), dimeric galectin-1 increases the amount of partially activated β1 integrins, but does not induce dimerization with α subunits (85). In the case of vascular smooth muscle cells, this interaction of galectin-1 with α1β1 integrin has been reported to both transiently phosphorylate focal adhesion kinase and modulate cell attachment, spreading and migration on laminin, but not on cellular fibronectin (38, 85). Thus galectin-1 is likely to affect smooth muscle cell adhesion by interacting with β1 integrin on the cell surface and inducing outside-in signaling (85) (Figure 1).

Galectin-1 also interacts with α5β1 integrin to restrict epithelial tumor cell growth (26) (Figure 1). Indeed, Fischer *et al* have observed that the anti-proliferative potential of galectin-1 in a number of carcinoma cell lines requires functional interaction with the α5β1 integrin (26) (Figure 1). Furthermore, we recently showed that the depletion of galectin-1 in various human glioma cell lines through both stable knockdown and transient targeted small-interfering RNA (siRNA) treatment induces an intracellular accumulation of integrin-β1 coincident with a diminution of integrin-β1 at points of cellular adhesion at the cell membrane, without altering the β1 gene expression level (28). Transient galectin-1 depletion effectuates as well the perinuclear accumulation of protein kinase C epsilon (PKCε) and the intermediate filament vimentin, both of which have been shown to promote integrin recycling in motile cells (28). These data argue for the involvement of galectin-1 in the PKCε/vimentin controlled trafficking of integrin-β1 (28) (Figure 1).

**Figure 2:**
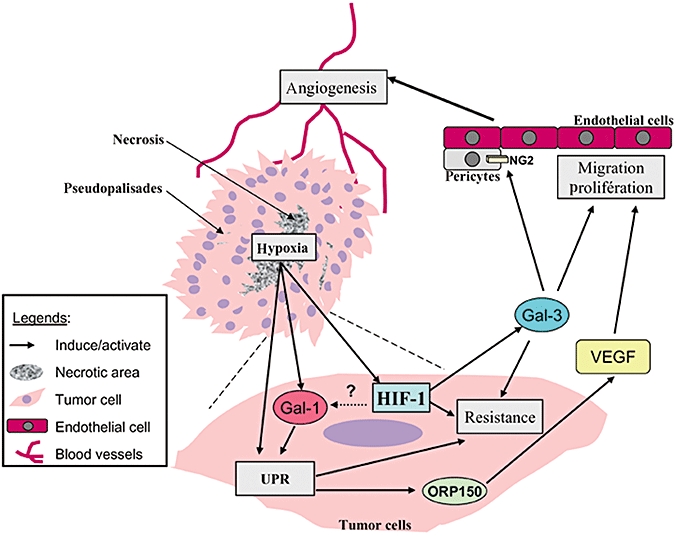
Presumed role of galectins in angiogenesis and chemoresistance. Necrotic foci in glioblastoma are typically surrounded by hypercellular zones referred to as pseudopalisades (104). It has been shown that pseudopalisades are hypoxic and express high amounts of the transcription factor hypoxia-inducible factor 1 (HIF-1) (104). Galectin-3 (Gal-3) expression is stimulated by hypoxia via HIF-1 (132) and stimulates angiogenesis *in vitro* and *in vivo*(89). Gal-3 has also been shown to interact with NG2 proteoglycan, a component of microvasculature pericytes, which stimulates endothelial cell motility and morphogenesis (130). In addition, Gal-3 is involved in chemoresistance, a process that is increased in hypoxic conditions (30). Galectin-1 (Gal-1) expression is also stimulated in hypoxic conditions (67), however, it is not known if this is HIF-1-dependent. Recent evidence indicates that Gal-1 plays an important role in angiogenesis and in chemo- and radioresistance (64, 120). These effects of Gal-1 can be explained at least partly by the fact that Gal-1 modulates the unfolded protein response (UPR), a process that is involved in resistance and angiogenesis (64, 65). Moreover, Gal-1 was shown to control the expression level of the UPR-regulated protein ORP150, which is responsible for vascular endothelial growth factor (VEGF) maturation and secretion (65).

Galectin-3 was also shown to bind to α1β1 integrin and it was suggested that this interaction regulates cell adhesion of various tumor cell lines by preventing α1β1 integrin interaction with the ECM proteins (94) (Figure 1). Galectin-3 also forms a complex with α3β1 integrins and NG2 on the surface of endothelial cells. The subsequent transmembrane signaling via α3β1 has been shown to be responsible for endothelial cell motility and angiogenesis (31) (Figures 1 and 2).

Finally, galectin-8 was also reported to interact with a subgroup of integrins that include α3β1, α6β1, and to a lesser extent with the α4 and the β3 subunits in human carcinoma (1299) cells (40). These interactions were shown to inhibit cell adhesion and to induce apoptosis (40). More recently, galectin-8 was shown to bind α1β1, α3β1 and α5β1 integrins in Jurkat T cells (14) (Figure 1).

As the aforementioned indicates, integrins are known to play a significant role in the malignant progression of cancer cell through their involvement in cell adhesion, motility and intracellular signaling (1, 41, 52), with an emphasis on the role of the beta 1 integrin subunit in gliomas (4, 6, 18, 95). As galectins bind integrins, with galectin-1, galectin-3 and galectin-8 all known specifically to modulate β1 integrin function, the understanding of molecular mediators such as galectins and the pathways through which they drive the cell invasion so descriptive of glioblastoma multiforme (GBM) is anticipated to reveal potential therapeutic targets that promote glioma malignancy (28). Indeed, targeting both integrins and galectins represents a feasible proposition in the future treatment of gliomas; already there is evidence amounting that attests to this. For example, the small α5β1 integrin antagonist, SJ749, reduces proliferation and clonogenicity of human astrocytoma cells (78). Moreover, impairing galectin-1 expression *in vivo* in experimental gliomas through the delivery of anti-galectin-1 siRNA augments the therapeutic benefits contributed by temozolomide (65).

## GALECTINS AND GLIOMA CELL MIGRATION

Cell migration involves at least three independent but highly coordinated biological processes: (i) cell adhesion to numerous components of the ECM; (ii) cell motility, which involves the reorganization of the actin cytoskeleton mainly through modification of the components of the adhesion complex; and (iii) invasion that involves the degradation of matrix proteins by tumor-secreted proteolytic enzymes, mainly serine proteases, cathepsins and metalloproteinases (MMPs) (21, 71, 103).

Galectins are involved in each of these steps (118). For example, galectin-1, galectin-3 and galectin-8 have been shown to influence glioma cell migration (10) (Figure 1). The expression of these galectins was shown to be higher in the invasive parts of xenografted glioblastomas than in the less invasive parts, suggesting their involvement in tumor astrocyte invasion of the brain parenchyma (10). In addition, galectin-1, galectin-3 and to a lesser extent galectin-8, markedly stimulate the migration of glioma cell lines (U373 and U87) *in vitro*(10) (Table 1). Moreover, galectin-3 biological functions were reported to be modulated by MMPs (93, 94), which play crucial roles in glioma cell motility and invasion (103). McClung *et al*(81) have shown by cDNA array analysis that secreted protein acidic and rich in cysteine, which is highly expressed in human gliomas and promotes glioma invasion, upregulates membrane type 1-matrix MMP and matrix MMP-2 transcripts, coincident with both increases in secreted galectin-3 and the proteolytically processed form of galectin-3. This concurrent stimulation of MMPs and galectin-3 supports a role for galectin-3 in glioma motility. However, in disagreement with the aforementioned, cultured galectin-3 deficient U373 glioblastoma cells, obtained by a stable transfection with a specific expression antisense plasmid, have been shown to both have increased motility potential on laminin and display modifications in cytoskeleton reorganization (20) (Table 1, Figure 1). c-DNA microarrays and quantitative immunofluorescence analysis showed that these galectin-3-deficient U373 cells have an increased expression of integrins-α6 and -β1 (20) (Figure 1). Although this study shows results that appear to contradict those of Camby *et al*, this could be partly explained by differences in the experimental procedures. Indeed, Debray *et al* have shown an increased motility of galectin-3 deficient cells cultivated on laminin (20) whereas Camby *et al* have observed an increase of motility when glioma cells were cultivated on plastic pre-coated with galectin-3 (10).

**Table 1:** Biological functions of galectins in gliomas. Abbreviation: CRD = carbohydrate-recognition domain.

Galectins	Structures	Cell types expressing and influenced by galectins in glioma context	Biological functions in gliomas	Presumed mechanism of action in glioma context	Intracellular (IC) or extracellular (EC) role	References
Galectin-1	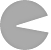	Astrocytes Oligodendrocyte Ependymocyte Endothelial cells Perivascular cells	Cell migration	•Modulation of cytoskeleton organization	EC	(11)
				•Modulation of RhoA expression	EC	(11)
				•Modulation of ADAM-15 expression	Unspecified	(12)
				•Modulation of integrin β1 recycling	Unspecified	(28)
			Angiogenesis	•Regulation of VEGF secretion via the regulation of ORP150 expression	Unspecified	(17, 65, 123)
			Chemo-/ Radioresistance	•Regulation of the endoplasmic reticulum stress response	Unspecified	(64, 120)
				•Modulation of p53 transcriptional activity	Unspecified	(64)
Galectin-3	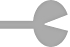	Astrocytes Oligodendrocytes Endothelial cells Perivascular cells Macrophages/ microglial cells	Cell migration	•Modulation of cytoskeleton organization	Unspecified	(20)
				•Modulation of integrins-α6 and -β1 expression	Unspecified	(20)
			Angiogenesis	•Interaction with NG2 proteoglycan on pericytes	EC	(36, 89)
Galectin-8	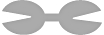	Astrocytes Perivascular cells	Cell migration	Unknown	EC	(10)

Our group has focused on the role of galectin-1 in glioma cell migration. We xenografted three human glioblastoma cell lines (H4, U87 and U373) into the brains of nude mice in order to characterize the *in vivo* galectin-1 expression pattern in relation to the tumor invasion of the normal brain parenchyma. The immunohistochemical analysis of galectin-1 expression in human U87 and U373 glioblastoma xenografts revealed a higher level of galectin-1 expression in invasive areas as compared to the non-invasive areas of the xenografts (11, 105). Moreover, nude mice intracranially grafted with U87 or U373 cells that were constitutively expressing low levels of galectin-1 (by stable transfection with an expression vector containing the antisense galectin-1 mRNA) had longer survival periods than those grafted with U87 or U373 cells unchanged in expression levels of galectin-1 (11). Complementary studies have shown that the *in vitro* addition of purified galectin-1 to U87 human GBM cells enhanced tumor cell motility in a lactose-inhibited manner (12) (Table 1). This effect appeared to be related to an increase in polymerized filamentous actin and the expression of the small guanosine triphosphatase RhoA (11) (Figure 1).

Finally, using cDNA microarray analysis and confirmation at protein levels, we observed that the U87 GBM cells that were galectin-1 deficient by means of an antisense galectin-1-stable transfection displayed increased protein levels for p21waf/cip1, cullin-2, p53, ADAM-15 and MAP-2 (12). Major differences in the expression patterns of ADAM-15 and in the actin stress fiber organization were also observed (12). The ADAM family of membrane-anchorage glycoproteins encompasses a catalytically active MMP domain and a disintegrin domain (98) and may thus be involved both in the proteolytic cleavage of cell-surface proteins and in integrin-mediated cell adhesion (including alpha9beta1 integrin/ADAM-15 interactions) via the RGD-dependent and -independent binding (25) (Figure 1).

All these data indicate that galectin-1 enhances the migratory capabilities of tumor astrocytes and, therefore, their biological aggressiveness. These features have been recently confirmed by Strik *et al*(120), and by Jung *et al*(57).

## GLIOMA, HYPOXIA, ANGIOGENESIS AND GALECTINS

Progression-associated genetic alterations are common to different glioma types, and target growth-promoting and cell-cycle control pathways resulting in focal hypoxia, necrosis and angiogenesis (76). GBM is distinguished pathologically from lower grade tumors by necrosis and microvascular hyperplasia (76). Necrotic foci are typically surrounded by “pseudopalisading” cells—a configuration that is relatively unique to malignant gliomas and has long been recognized as an ominous prognostic feature (Figure 2) (76, 104). Recent investigations have demonstrated that pseudopalisades are severely hypoxic, overexpress hypoxia-inducible factor 1 (HIF-1), and secrete proangiogenic factors such as vascular endothelial growth factor (VEGF) and IL-8 (104) (Figure 2). Pseudopalisades could represent a wave of tumor cells actively migrating away from central hypoxia that arises after a vascular insult (104). HIF-1 is one of the master regulators that orchestrate the cellular responses to hypoxia; it is a heterodimeric transcription factor composed of alpha and beta subunits (58). The alpha subunit is stable in hypoxic conditions but is rapidly degraded in normoxia; upon stabilization or activation, HIF-1 translocates to the nucleus and induces transcription of its downstream target genes (58). Most relevant to gliomagenesis, HIF-1 is a potent activator of angiogenesis and invasion through its upregulation of target genes critical for these functions; activation of the HIF-1 pathway is a common feature of gliomas and may explain the intense vascular hyperplasia often seen in GBM (27, 32, 58).

The expression of galectin-3, which is the most extensively studied member of the galectin family in the field of angiogenesis (89, 125) and with specific regard to gliomas (36, 130) (Table 1), has been shown to be stimulated upon HIF-1 activation (37, 50, 117, 132) (Figure 2). It has been shown that galectin-3 promotes chemotaxis and morphogenesis of endothelial cells and stimulates capillary tube formation in human umbilical vein endothelial cells *in vitro*(89). Moreover, galectin-3 was also reported to stimulate angiogenesis *in vivo* in breast cancer cell lines injected subcutaneously into the dorsolateral region of nude mice (89). Galectin-3 was also shown to interact with NG2 proteoglycan, a component of microvascular pericytes, which stimulates endothelial cell motility and morphogenesis. This function of NG2 on cell motility and morphogenesis depends on the formation of a complex with galectin-3 and α3β1 integrin to stimulate integrin-mediated transmembrane signaling (31, 130) (Figure 2).

Galectin-1 is also a hypoxia-regulated protein (7, 67, 68) that has been shown recently to display major roles in angiogenesis (17, 123), in both gliomas (65) (Table 1) and melanomas (80) (Figure 2). Galectin-1 involvement in tumor angiogenesis was first suggested after the discovery that both vascular smooth muscle and endothelial cells express the protein (84). Clausse *et al* had also previously shown that galectin-1 was upregulated in capillaries associated with carcinoma cells and found that galectin-1 could mediate interactions between tumors and endothelial cells *in vitro*, suggesting a potential role for galectin-1 in modulating angiogenesis (17). Finally, Thijssen *et al* have shown that both treatment with galectin-1 specific antisense oligodeoxynucleotides or with polyclonal anti-galectin-1 antibodies resulted in inhibition of endothelial cell proliferation and migration, demonstrating an essential role for galectin-1 during angiogenesis (123). The role of galectin-1 in tumor angiogenesis is further highlighted in galectin-1-null mice, in which tumor growth is markedly impaired because of insufficient tumor angiogenesis (123).

We ourselves have also put forward evidence for the role of galectin-1 in the process of angiogenesis using human glioma cells. To determine how galectin-1 exerts its pro-angiogenic effects, we investigated galectin-1 signaling in the human Hs683 glioma cell line. We observed that galectin-1 signals through the endoplasmic reticulum transmembrane kinase/ribonuclease inositol-requiring 1alpha (IRE1alpha) that regulates the expression of oxygen-regulated protein 150 (ORP150), which in turn controls VEGF maturation (Figure 2). Galectin-1 also modulates the expression of six other hypoxia-related genes (ie, *CTGF*,*ATF3*, *PPP1R15A*, *HSPA5*, *TRA1* and *CYR61*) that are implicated in angiogenesis (65). Moreover, we have recently reported that downregulating galectin-1 expression in Hs683 human glioma cells through targeted siRNA provokes a marked decrease in the expression of the brain expressed X-linked gene (BEX2), a feature which confers increased survival in Hs683 orthotopic xenograft-bearing mice. This decrease in BEX2 expression impairs vasculogenic mimicry channel formation *in vitro* and angiogenesis *in vivo*, and modulates glioma cell adhesion, motility and invasive features (66).

## GLIOMA HYPOXIA, CHEMO-/RADIORESISTANCE AND GALECTINS

Resistance of human tumors to anticancer drugs is most often ascribed to gene mutations, gene amplification or epigenetic changes that influence the uptake, metabolism or export of drugs from single cells (126). Another important yet little-appreciated cause of anticancer drug resistance is the limited ability of drugs to penetrate tumor tissue and to reach all of the tumor cells in a potentially lethal concentration (126). Although now recognized as a major contributor to cancer malignant progression and to treatment failure, the precise role of hypoxia signaling in cancer and in prognosis still needs to be further defined (8). As emphasized earlier, intra-tumoral hypoxia causes genetic changes in malignant gliomas that produce a microenvironment that selects for cells of a more aggressive phenotype (55, 104). Hypoxia can initiate cell demise by apoptosis/necrosis but also prevent cell death by provoking adaptive responses that, in turn, facilitate cell proliferation or angiogenesis, thus contributing to tumor malignant progression (133). Zhou J *et al*(133) emphasize that considering that activation of HIF-1 provokes pro-survival as well as pro-death decisions under hypoxia, it will be crucial to understand decision making processes in regulating cell death, adaptation and chemoresistance. Hypoxia is also known to modulate the unfolded protein response, a coordinated program that promotes cell survival under conditions of endoplasmic reticulum (ER) stress (60), and which is known to contribute to tumor malignant progression and drug resistance of solid tumors (59).

As mentioned earlier, hypoxia is known to activate galectin-1 expression (7, 67, 68), and galectin-1 was found to be negatively regulated by transfection with TP53 in glioma cells (99).

We recently reported that temozolomide, the standard treatment for glioma patients (74), increases galectin-1 expression in Hs683 glioma cells both *in vitro* and *in vivo*. In addition, galectin-1 expression was shown to be upregulated by ionizing irradiation of glioma cell lines *in vitro*(120). Reducing galectin-1 expression in these cells by siRNA increases the antitumor effects of various chemotherapeutic agents, in particular temozolomide both *in vitro* and *in vivo* in a orthotopic xenograft mouse model (64) (Table 1, Figure 2). We also observed that decreasing galectin-1 expression by means of an anti-galectin-1 siRNA in the mouse B16F10 metastatic melanoma model, which is a syngenic model whereby B16F10 melanoma cells are injected in the tail vein, also increases the therapeutic benefits contributed by temozolomide *in vivo*(80). This decrease of galectin-1 expression in the B16F10 mouse melanoma cells does not modify their sensitivity to apoptosis nor autophagy. However, it does induce heat shock protein 70-mediated lysosomal membrane permeabilization (LMP), a process associated with cathepsin B release into the cytosol, which in turn is believed to sensitize the cells to the proautophagic effects of temozolomide when grafted *in vivo*(80). In Hs683 glioma cells a decrease in galectin-1 expression does not induce apoptotic or autophagic features and does not induce LMP, but is found to modulate p53 transcriptional activity and decrease p53-targeted gene expression including DDIT3/GADD153/CHOP, DUSP5, ATF3 and GADD45A (64). In addition the decrease in galectin-1 expression impairs the ER stress response, which is believed to play a role in drug resistance (79), and also impairs the expression levels of seven other genes implicated in chemoresistance: *ORP150*; *HERP*; *GRP78/Bip*; *TRA1*; *BNIP3*; *GADD45B*; and *CYR61*, some of which are also known to be modified by hypoxia (64).

Similar to galectin-1, galectin-3 is also a hypoxia-regulated protein (37, 50, 117, 132) that is implicated in cancer drug resistance (29, 30) (Figure 2). Galectin-3 confers chemoresistance to a wide variety of cancer cell types (30). Recent studies have revealed that galectin-3 demonstrates anti-apoptotic effects, which contribute to cell survival in several types of cancer cells (88). In particular, intracellular galectin-3, which contains the NWGR anti-death motif of the Bcl-2 family, inhibits cell apoptosis induced by chemotherapeutic agents such as cisplatin and etoposide in some types of cancer cells (88). It has also been reported that the nuclear export of phosphorylated galectin-3 regulates its anti-apoptotic activity in response to chemotherapeutic drugs. Indeed anticancer drugs can induce DNA damage, which causes phosphorylated galectin-3 to translocate from the nucleus to the cytoplasm and regulates phosphorylation of Bad, Akt and extracellular signal-regulated kinase resulting in stabilization of mitochondrial membrane integrity. The stabilization of the mitochondrial membrane prevents cytochrome *c* release and subsequent caspase activation, resulting in the suppression of apoptosis and anticancer drug resistance (30). Finally, it has been suggested that targeting galectin-3 could improve the efficacy of anticancer drug chemotherapy in several types of cancer (30). However, the involvement of galectin-3 in the chemo- and/or radioresistance of gliomas has not yet been demonstrated, at least to the best of our knowledge.

## CONCLUSIONS

Galectins are known to play an important role in cancer malignant progression (75). Specifically, galectin-1, galectin-3 and to a lesser extent galectin-8 have been reported to be implicated in glioma malignant progression (Table 1). Galectin-1 especially is involved in many different steps of tumor biology, such as migration, angiogenesis and resistance to chemotherapy and radiotherapy. Moreover, galectin-1 is also involved in tumor immune escape, however not in gliomas (96, 102). Although there is much to learn about the actual mechanisms by which galectins influence glioma cell biology and much to explain regarding some conflicting results obtained in different studies, the data reviewed here may be amenable to therapeutic manipulation. We have already shown that decreasing galectin-1 expression in human GBM orthotopic xenografts in mouse brains by siRNA administration enhances the therapeutic benefits of temozolomide (65). Thus, galectins and especially galectin-1 could be important targets for the development of new anticancer drugs not only for gliomas but for other types of cancer as well (53, 100).
